# Improved evaluation of waveform reconstruction in speech decoding based on invasive brain-computer interfaces

**DOI:** 10.1162/IMAG.a.146

**Published:** 2025-09-10

**Authors:** Xiaolong Wu, Kejia Hu, Zhichun Fu, Dingguo Zhang

**Affiliations:** Department of Electronic and Electrical Engineering, University of Bath, Bath, United Kingdom; Department of Neurosurgery, Center for Functional Neurosurgery, Ruijin Hospital Affiliated with Shanghai Jiao Tong University School of Medicine, Shanghai, China

**Keywords:** intracranial signals, brain-computer interface (BCI), speech decoding, speech prosthesis, evaluation method

## Abstract

Brain-computer interfaces (BCIs) that reconstruct speech waveforms from neural signals are a promising communication technology. However, the field lacks a standardized evaluation metric, making it difficult to compare results across studies. Existing objective metrics, such as correlation coefficient (CC) and mel cepstral distortion (MCD), are often used inconsistently and have intrinsic limitations. This study addresses the critical need for a robust and validated method for evaluating reconstructed waveform quality. Literature about waveform reconstruction from intracranial signals is reviewed, and issues with evaluation methods are presented. We collated reconstructed audio from 10 published speech BCI studies and collected Mean Opinion Scores (MOS) from human raters to serve as a perceptual ground truth. We then systematically evaluated how well combinations of existing objective metrics (STOI and MCD) could predict these MOS scores. To ensure robustness and generalizability, we employed a rigorous leave-one-dataset-out cross-validation scheme and compared multiple models, including linear and non-linear regressors. This work, for the first time, identifies a lack of a standard evaluation method, which prohibits cross-study comparison. Using 10 public datasets, our analysis reveals that a non-linear model, specifically a Random Forest regressor, provides the most accurate and reliable prediction of subjective MOS ratings (R² = 0.892). We propose this cross-validated Random Forest model, which maps STOI and MCD to a predicted MOS score, as a standardized objective evaluation metric for the speech BCI field. Its demonstrated accuracy and robust validation outperform the available methods. Moreover, it can provide the community with a reliable tool to benchmark performance, facilitate meaningful cross-study comparisons for the first time, and accelerate progress in speech neuroprosthetics.

## 1 Introduction

Brain-computer interface (BCI) technology has emerged as a transformative tool to interact with the external environment by only thoughts (Brandman et al., 2017; Parvizi & Kastner, 2018; Volkova et al., 2019; Wu et al., 2024). By decoding brain signals, BCIs enable individuals to interact with computers or control devices, offering significant applications in assistive technology, rehabilitation, and communication. Among various BCI paradigms, speech BCIs focus on the reconstruction or decoding of speech-related information from neural signals (Cooney et al., 2018, 2022; Luo et al., 2022; Martin et al., 2018, 2019; Rabbani et al., 2019; Silva et al., 2024). This field has gained momentum due to its potential to restore communication for individuals with speech impairments.

Speech BCI decoding targets can be broadly categorized into two approaches: decoding text (Card et al., 2024; Duraivel et al., 2023; Luo et al., 2023; Makin et al., 2020; Metzger et al., 2022, 2023; Moses et al., 2021; Proix et al., 2022; Sun et al., 2020; Willett et al., 2021, 2023; Wilson et al., 2020; Zhang et al., 2024) and reconstructing waveforms (Akbari et al., 2019; Angrick, Ottenhoff, Goulis, et al., 2021; Angrick, Ottenhoff, Diener, et al., 2021; Angrick et al., 2019, 2024; Anumanchipalli et al., 2019; Berezutskaya et al., 2022; Chen et al., 2024; Herff et al., 2019; Kohler et al., 2021; Liu et al., 2023; Verwoert et al., 2022; Wairagkar et al., 2023; Wilson et al., 2020; Wu et al., 2024). Text decoding translates neural signals into textual representations, providing a direct means of communication. In contrast, waveform reconstruction aims to regenerate audio signals that capture the speaker’s voice characteristics, intonation, and other paralinguistic speech features. While waveform reconstruction is particularly challenging due to the complexity of mapping neural signals to high-dimensional acoustic representations, it holds promise for enabling more naturalistic and expressive communication.

**Fig. 1.**
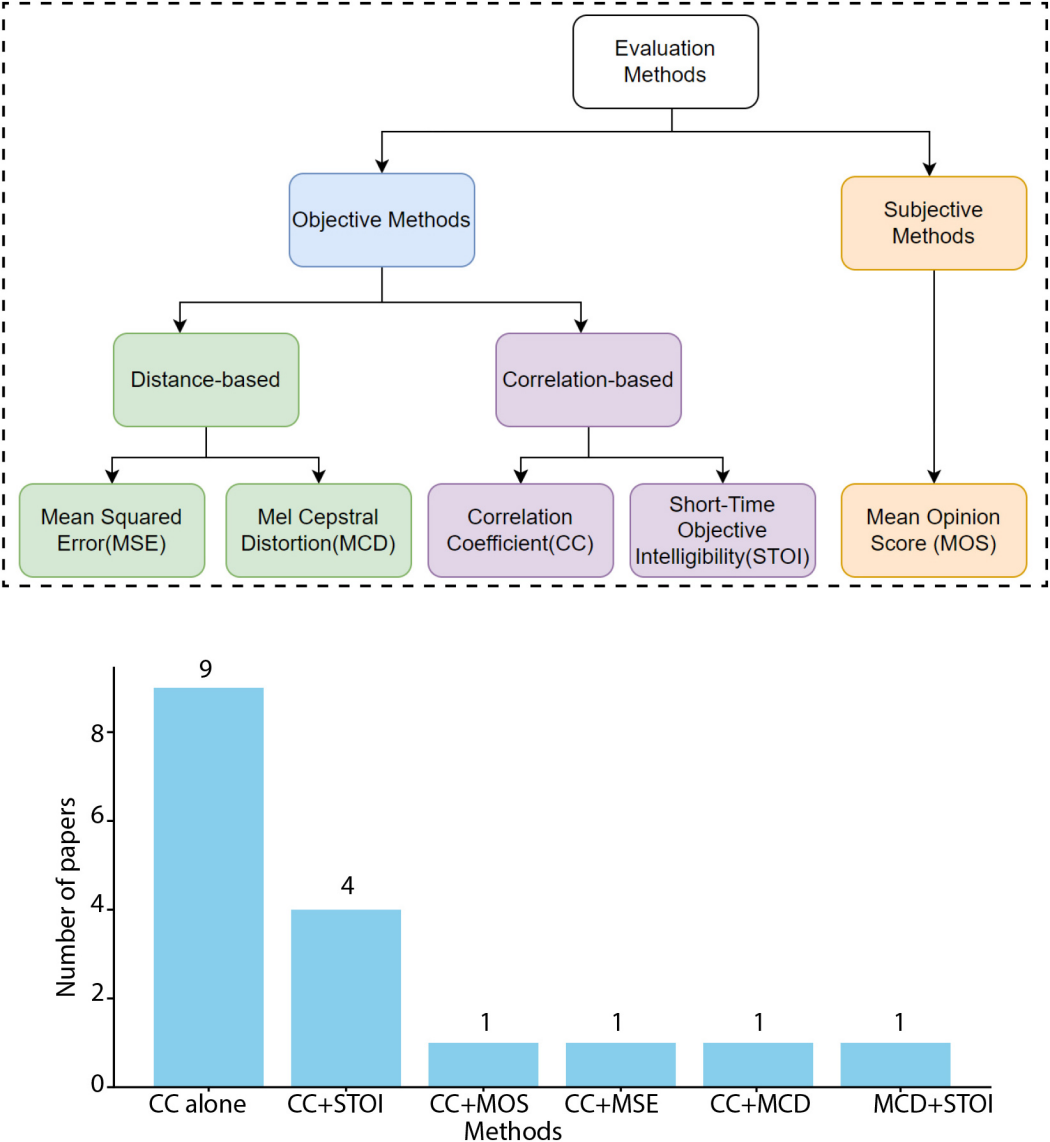
Evaluation methods and their usage in the literature. The upper subplot describes all methods, while the lower subplot shows the number of studies using various methods or method combinations. Note that almost all existing studies solely used CC as the evaluation method, which could be misleading and prohibit the comparison between studies.

For waveform reconstruction, because spectrograms capture essential audio features, including frequency content and temporal variations, almost all existing pieces of the literature evaluate their decoding performance by comparing spectrograms of the original and the reconstructed waveforms. These evaluation methods can be divided into two main categories: objective and subjective tests. Further, objective measurements can be further categorized into two types: distance-based measurement, which includes mean squared error (MSE) and mel cepstral distortion (MCD), and correlation-based measurement, which includes correlation coefficient (CC) and short-time objective intelligibility (STOI). Figure 1 is produced to obtain an overall view of the existing evaluation methods and their current usage in the literature. The upper subplot describes all methods, while the lower subplot shows the number of studies using various methods or method combinations. This plot shows that CC was mostly used in the literature, and 9 out of 16 studies used CC alone, which could be problematic because of issues with CC presented in the main sections.

The following contents review existing studies of waveform reconstruction using various evaluation methods.

### 1.1 Objective measurements

Objective evaluation methods quantify the performance of waveform reconstruction using mathematical and statistical metrics. Existing objective measurements can be categorized into two classes: distance-based measurement and correlation-based measurement.

*1) Distance-based Measurements:* Distance-based measurements quantify the dissimilarity between two signals by calculating the “distance” between their corresponding values. Common distance-based metrics include Euclidean distance (L2 norm distance), which measures the root of squared differences, and Manhattan distance, which sums absolute differences (L1 norm distance). In the speech BCIs literature, there are two popular distance-based measurements used: mean squared error (MSE) and mel cepstral distortion (MCD).

MSE calculates the average squared difference between the spectrogram of the original and reconstructed waveforms, providing a measure of reconstruction accuracy. This method has been used in a recent study where MSE ranges from approximately 1.0 to 4.7 were obtained using sEEG signals for 10 epileptic patients (Wu et al., 2024).

MCD evaluates the distortion in the spectral envelope of the reconstructed waveform. This method has been used in an ECoG study and obtained 5.14 dB to 6.58 dB (Anumanchipalli et al., 2019).

*2) Correlation-based Measurement:* Correlation-based measurements assess the similarity between two signals by evaluating how well they co-vary over time, capturing their shared patterns or trends. Metrics like the Pearson correlation coefficient measure the linear relationship, ranging from -1 (perfectly inversely correlated) to 1 (perfectly correlated), while a value near 0 indicates no linear correlation. These methods are less sensitive to amplitude differences and shifts but focus on the co-oscillation between signals. Correlation-based metrics are particularly useful for identifying relationships between signals that share similar shapes but differ in scale or offset.

In the literature, two such measurements are used: correlation coefficient (CC) (Angrick, Ottenhoff, Goulis, et al., 2021; Angrick, Ottenhoff, Diener, et al., 2021; Angrick et al., 2019, 2024; Anumanchipalli et al., 2019; Berezutskaya et al., 2022; Chen et al., 2024; Herff et al., 2019; Kohler et al., 2021; Liu et al., 2023; Verwoert et al., 2022; Wairagkar et al., 2023; Wilson et al., 2020; Wu et al., 2024) and short-time objective intelligibility (STOI) (Akbari et al., 2019; Angrick et al., 2019; Berezutskaya et al., 2022; Herff et al., 2019).

CC quantifies the linear relationship between the original and reconstructed waveforms, indicating the degree of similarity. It has been heavily used in the literature. For example, CC scores ranging from approximately 0.4 to 0.9 were obtained in a sEEG study (Verwoert et al., 2022). In another study, a CC score of 0.83 was obtained using ECoG signals (Chen et al., 2024). In an MEA (micro-electrode array) study, Wilson et al. (2020) obtained an average CC score of 0.52 from two patients with spinal cord injury (SCI).

The short-time objective intelligibility (STOI) metric improves upon the correlation coefficient (CC) by providing a more detailed, perceptually relevant assessment of signal similarity, particularly for speech intelligibility. Unlike the correlation coefficient, which measures linear relationships globally, STOI analyzes short-time segments to account for time-varying distortions and aligns with human auditory perception. This makes STOI more robust to non-linear distortions, noise, and time misalignments, offering better alignment with subjective listening tests for speech quality and intelligibility evaluation. Despite that, very few studies utilized this method, one of which obtained STOI scores approximately ranging from 0.1 to 0.4 from four patients with epilepsy and one patient with a brain tumor (Berezutskaya et al., 2022). In another ECoG, Angrick et al. (2019) obtained STOI ranging from approximately 0.2 to 0.5 using an advanced deep learning method from six patients who underwent awake craniotomies for brain tumor resection. Other studies used a combination of STOI and CC (Angrick et al., 2019; Berezutskaya et al., 2022; Herff et al., 2019), and a combination of STOI and MOS (Akbari et al., 2019).

### 1.2 Subjective measurements

Subjective evaluation methods rely on human judgment to assess the perceptual quality and intelligibility of the reconstructed waveforms. The most popular subjective measurement of waveform reconstruction in speech BCIs is the mean opinion score (MOS). MOS measures perceived quality by collecting subjective ratings from human evaluators. In this method, participants rate test signals, such as audio or video, on a 5-point scale ranging from “Bad” to “Excellent” in a controlled environment. The MOS is calculated as the average of all participants’ scores for each signal, providing an overall perception-based quality measure. This method has been used in combination with CC in Liu et al. (2023), and in combination with STOI in (Akbari et al., 2019).

Subjective tests are time-consuming and may introduce variability due to listener bias. On the other hand, objective tests are necessary as they provide standardized and reproducible metrics, enabling researchers to quantitatively assess and compare reconstruction performance without the influence of subjective variability. Therefore, in this study, we will only focus on the objective metrics and try to propose an optimal objective measurement.

### 1.3 Limitations with current studies

Existing studies use different evaluation methods, or the same evaluation method with different parameters, which makes it impossible to do a cross-study comparison

First, different evaluation methods prevent a cross-study comparison. For example, a combination of STOI and MOS was used in one study (Akbari et al., 2019), while CC alone was used in other studies (Verwoert et al., 2022; Wu et al., 2024). Various evaluation methods used in existing studies are presented in Figure 1. In this figure, it is clear that different studies used very different evaluation methods, and it is impossible to perform a cross-study comparison.

In addition, the cross-study comparison is still difficult even if the same method is used but with different parameters. For example, CC was used in studies based on sEEG (Verwoert et al., 2022; Wu et al., 2024), ECoG (Chen et al., 2024) and MEA (Wilson et al., 2020). While a higher CC score was obtained in these two sEEG studies, the subject evaluation of the reconstructed waveform reveals that the reconstruction result is better for the latter two studies. This discrepancy is caused by a different parameter: the time window in the first two studies is longer than that in the latter two.

### 1.4 Limitations with individual method

Despite the fact that numerous waveform reconstruction studies exist and the above-mentioned evaluation methods are heavily used in these studies, we have identified several limitations with the existing evaluation methods.

- **CC** Correlation coefficient (CC) assesses how two signals co-vary with each other. However, CC primarily focuses on the relative trajectory trend of two signals rather than their absolute differences, highlighting its limitation in capturing absolute spectral or amplitude discrepancies, such as those reflected in Euclidean distance, limiting their utility in fully evaluating reconstruction performance. Another equally important criticism of CC is that the CC scores can be significantly different if different window sizes are used.
- **STOI** Short-time objective intelligibility (STOI) is a widely used objective speech quality measure. However, similar to CC, it is also a correlation-based measurement and has intrinsic limitations associated with the correlation-based measurement, such as the inability to capture absolute differences and inconsistency caused by using different signal lengths.
  Having said that, STOI is preferred compared to CC because of the usage of a small window, which could mitigate the inconsistency caused by different signal lengths.
- **MSE** Mean squared error (MSE) evaluates overall reconstruction accuracy but lacks perceptual relevance and does not reflect intelligibility or speaker identity fidelity.
- **MCD** Although MCD assesses spectral envelope distortion, it does not account for perceptual intelligibility or temporal alignment issues as in STOI. Having said that, MCD is a preferred distance-based measurement compared to MSE because it operates on mel-frequency cepstral coefficients (MFCC) features of the waveform, which correspond well to the human auditory system.

Due to the intrinsic shortcomings associated with individual methods, it can be misleading or erroneous to use only one method or a suboptimal combination. To obtain an overall view of the current usage of these existing methods, a bar plot was produced by counting paper numbers using different methods in Figure 1. This plot shows that 9 out of 17 studies on speech waveform reconstruction based on the intracranial signals use CC as the evaluation method alone. Considering the issues with CC, this could complicate the comparison between studies and confuse the research community. Although this confusion was mitigated in the other 5 studies by combining CC with other methods, the chosen combinations are still suboptimal. From the previous discussion in the introduction, there are two categories of objective methods, distance-based and correlation-based measurements, and it is necessary to combine these two categories to obtain a comprehensive evaluation. According to the previous discussion in the limitation section I-D, for the correlation-based category, STOI is preferred to CC, and MCD is preferred to MSE for a distance-based category. Therefore, the optimal objective combination should be STOI and MCD. However, none of the studies in Figure 1 employed such a combination.

Considering the previous issues in the existing literature, in this work, we propose to use a combination of STOI and MCD as the optimal evaluation method of waveform reconstruction from the intracranial signals. To support our proposal, 10 datasets are used in this study, taken from 10 waveform reconstruction studies covering sEEG, ECoG, and MEA signals. In these 10 studies, either the code and data were released, and the waveform could be reconstructed, or both the original and reconstructed waveforms were released.

### 1.5 Novelty

In summary, despite many studies that have been conducted in waveform reconstruction from intracranial signals, no paper has tried to investigate the issues in the employed evaluation methods, and there is a lack of a standard evaluation measurement in this line of research.

In this paper, we aim to achieve two goals: one is to identify the shortcomings of the existing objective evaluation methods, and the second is to propose an optimal objective evaluation method.

The novelties in this study are:

1. Identifying critical issues in current studies using correlation-based and distance-based measurement.
2. Proposing an optimal objective evaluation method combining STOI and MCD that outperforms the available methods.
3. For the first time, this work enables cross-study comparison using the proposed objective method. Before this study, it was impossible to compare the existing studies due to the different methods and parameters used in the existing literature.

By employing available public datasets from 10 studies, this work contributes to the development of more reliable and comparable methods for assessing waveform reconstruction performance in speech BCIs.

## 2 Data and Methods

### 2.1 Data description

Ten datasets were used in this study, including studies using stereo-electroencephalography (sEEG), electrocorticography (ECoG), and multi-electrode array (MEA). In these 10 datasets, one study (study 1) released the sEEG data and code, while the other nine studies released the reconstructed audio files. Table 1 provides a summary of the datasets used. Below is the detailed information regarding these 10 studies.

**Table 1.** Summary of datasets used in this study.

Dataset ID	Modality	Task type	# of samples	Avg. duration (s)
1 (Verwoert et al., 2022)	sEEG	Single words	19**	2.2
2 (Kohler et al., 2021)	sEEG	Sentences	8*	3.4
3 (Wilson et al., 2020)	MEA	Single words	6*	2.6
4 (Chen et al., 2024)	ECoG	Single words	7*	1.5
5 (Herff et al., 2019)	ECoG	Single words	7*	2.1
6 (Angrick et al., 2019)	ECoG	Single words	8	2.6
7 (Liu et al., 2023)	ECoG	Tonal words	8*	1.7
8 (Berezutskaya et al., 2022)	ECoG	Single words	8	1.3
9 (Anumanchipalli et al., 2019)	ECoG	Sentences	4*	3.4
10 (Akbari et al., 2019)	ECoG	Perceived speech	4*	2.8

* Audio samples provided in the original work were split into short intervals to increase dataset size.

** The audio samples are generated using methods described in their paper.

1) Dataset 1 (sEEG) (Verwoert et al., 2022). Ten epileptic patients implanted with depth electrodes (native speakers of Dutch) were recruited in this sEEG study. Participants were required to read aloud words that were shown to them on a laptop screen. One random word from the stimulus library (the Dutch IFA corpus (van Son et al., 2001) extended with the numbers 1 to 10 in word form) was presented on the screen for a duration of 2 s during which the participant read the word aloud once, during which the neural and audio signals were recorded simultaneously.

2) Dataset 2 (sEEG) (Kohler et al., 2021). Three patients (P1, 16 y/o male; P2, 20 y/o female; P3, 40 y/o male) suffering from intractable epilepsy were recruited in this study, and all were native speakers of Dutch. Patients were implanted with depth electrodes to identify the epileptic foci and plan potential resections. During the experiment, a total of 100 sentences (between 5 and 7 words long) from the Mozilla Common Voice Dutch corpus (Ardila et al., 2020) were displayed on a laptop in pseudo-randomized order. Each sentence was followed by a 2-s rest interval during which a fixation cross was shown on the screen.

3) Dataset 3 (MEA) (Wilson et al., 2020). Two patients with spinal cord injury from BrainGate2 were recruited in this study, both of whom were implanted with the MEA electrodes. The participants were required to make speech production of individual words as they were displayed. There are a total of 420 unique words that widely sample American English phonemes.

4) Dataset 4 (ECoG) (Chen et al., 2024). There are 48 native English speakers recruited in this study, all of whom are patients with refractory epilepsy who had ECoG subdural electrode grids implanted. A total of 50 English words were used in this study and the patients were required to speak aloud in three tasks: auditory repetition (AR, repeating auditory words), auditory naming (AN, naming a word based on an auditory definition), sentence completion (SC, completing the last word of an auditory sentence), visual reading (VR, reading aloud written words) and picture naming (PN, naming a word based on a color drawing).

5) Dataset 5 (ECoG) (Herff et al., 2019). Six patients, native English speakers, undergoing awake craniotomy with cortical stimulation and recording as part of normal clinical care, were recruited in this study. During the experiment, participants were required to read aloud words displayed on a laptop, most of which were monosyllabic and followed a consonant-vowel-consonant (CVC) structure. These words were taken from the Modified Rhyme Test (House et al., 1963) and supplemented with additional words to better reflect the phoneme distribution of American English.

6) Dataset 6 (ECoG) (Angrick et al., 2019). ECoG from six native English-speaking participants while they underwent awake craniotomies for brain tumor resection. During the experiment, participants were required to read aloud between 244 and 372 words displayed on a laptop, most of which were monosyllabic and followed a consonant-vowel-consonant (CVC) structure.

7) Dataset 7 (ECoG) (Liu et al., 2023). There are five native Chinese-speaking patients (four males aged 44, 53, 39, and 54 and one female aged 37) who underwent awake language mapping during their brain tumor surgeries and were recruited. In this tonal language (Chinese) study, participants were required to read aloud 2 Chinese words in four different tones, resulting in eight different syllables. This study aimed to synthesize speech in a tonal language from invasive neural recordings using high-density ECoG.

8) Dataset 8 (ECoG) (Berezutskaya et al., 2022). Four patients with medication-resistant epilepsy were recruited in this study after they were implanted with subdural ECoG electrode grids to determine the source of seizures and test the possibility of surgical removal of the corresponding brain tissue. Twelve unique Dutch words were used in this study, which were taken from the Dutch children’s book ‘Jip and Janneke’. All words were presented in random order, and the participants were required to read them aloud.

9) Dataset 9 (ECoG) (Anumanchipalli et al., 2019). Five participants who underwent chronic implantation of a high-density, subdural electrode array over the lateral surface of the brain as part of their clinical treatment for epilepsy were enrolled in this study. Participants were required to read aloud sentences. There are a total of 460-730 sentences taken from the MOCHA-TIMIT (“MOCHA-TIMIT”, n.d.), and several books (Sleeping Beauty, Frog Prince, Hare and the Tortoise, The Princess and the Pea, and Alice in Wonderland).

10) Dataset 10 (ECoG) (Akbari et al., 2019). Five patients with pharmacoresistant focal epilepsy were recruited, all of whom underwent chronic intracranial encephalography (iEEG) implantation to identify epileptogenic foci in the brain for later removal. The goal of this study was to reconstruct the perceived speech uttered by others. The speech materials included continuous speech stories and 10 digits (zero to nine), uttered by four voice actors and actresses.

### 2.2 Evaluation methods

Five methods are used in the existing literature, including MOS, STOI, CC, MSE, and MCD. However, MSE is excluded from this study because MCD is a better distance-based candidate for audio quality evaluation. We prioritized MCD due to its perceptual relevance, as it operates on mel-frequency cepstral coefficients (MFCCs) which are derived from a model of the human auditory system’s perception of sound, and its prevalence as a standard metric in speech synthesis literature. Therefore, four methods are evaluated in this work: MOS, STOI, CC, and MCD, and their detailed information is presented below.

*1) Mean Opinion Scores (MOS):* The Mean Opinion Score (MOS) is a subjective evaluation metric widely used to measure the perceived quality of audio, video, or multimedia systems. It is based on structured listening or viewing tests where human subjects rate the quality on a predefined scale, typically from 1 (bad) to 5 (excellent). The final MOS is computed as the arithmetic mean of all individual scores, offering a numerical representation of overall quality. This metric is particularly valuable in assessing systems such as speech synthesis, audio enhancement, and telecommunications, as it reflects real user experiences.

The Mean Opinion Score (MOS) is calculated as:

$$\begin{array}{cc} \text{MOS} & =\frac{1}{N}\sum_{i=1}^{N}R_{i} \end{array}$$ (1)

where:

- $R_{i}$ is the quality rating provided by the i-th participant,
- N is the total number of participants.

To obtain a comprehensive subjective evaluation of these datasets, 14 evaluators were recruited (either native English speakers or fluent in English). Each evaluator listened to the original audio first, then the reconstructed audio, and provided a score. Each trial was rated three times in a randomized order. To assess the consistency of the subjective ratings, we calculated the inter-rater reliability using Cronbach’s alpha. The resulting value of 0.89 indicates a high degree of reliability among the raters, justifying the use of the aggregated MOS scores as a stable ground-truth metric.

*2) Short-Time Objective Intelligibility (STOI):* The short-time objective intelligibility (STOI) score is calculated as follows:

$$\begin{array}{cc} \text{STOI}\left(s,\hat{s}\right) & =\frac{1}{N}\sum_{j=1}^{N}\text{corr}(y_{j},{\hat{y}}_{j}) \end{array}$$ (2)

where:

- s is the clean speech signal,
- $\hat{s}$ is the degraded or processed speech signal,
- ${\text{y}}_{j}$ and ${\text{y}}_{j}$ are MFCCs representations of s and $\hat{s}$ , respectively.
- $\left(y_{j},{\hat{y}}_{j}\right)$ computes the linear correlation coefficient between ${\text{y}}_{j}$ and ${\hat{y}}_{j}$ ,
- N is the total number of short-time segments used in the analysis.

*3) Correlation Coefficient (CC):* The formula for the Pearson correlation coefficient is given by:

$$\begin{array}{cc} r & =\frac{\sum_{i=1}^{n}\left(x_{i}-\bar{x}\right)\left(y_{i}-\bar{y}\right)}{\sqrt{\sum_{i=1}^{n}{\left(x_{i}-\bar{x}\right)}^{2}}\sqrt{\sum_{i=1}^{n}{\left(y_{i}-\bar{y}\right)}^{2}}} \end{array}$$ (3)

where:

- $x_{i}$ and $y_{i}$ are the individual data points of variables x and y,
- $\bar{x}$ and $\bar{y}$ are the means of x and y, respectively,
- n is the total number of data points.

Both STOI and CC were calculated on the MFCC feature of the original and reconstructed waveforms. Our choice to compute STOI and CC over the entire utterance, including silent periods, was a deliberate one grounded in the specific requirements of a speech BCI system. A system’s ability to correctly generate silence (i.e., accurate voice activity detection) is a critical component of its real-world utility. By including silence, this metric appropriately penalizes systems that generate spurious noise during non-speech intervals, thus providing a more realistic measure of total system performance.

*4) Mel Cepstral Distortion (MCD):* The MCD is calculated as the Euclidean distance between the mel-frequency cepstral coefficients (MFCCs) of the original and synthesized speech, and it is calculated as follows:

$$\begin{array}{cc} \text{MCD} & =\frac{10}{\text{ln}\left(10\right)}\sqrt{2}\cdot \frac{1}{T}\sum_{t=1}^{T}\sqrt{\sum_{m=1}^{M}{\left(c_{m}\left(t\right)-{\hat{c}}_{m}\left(t\right)\right)}^{2}} \end{array}$$ (4)

where:

- $c_{m}\left(t\right)$ are the mel-frequency cepstral coefficients (MFCCs) of the original speech at time frame t,
- ${\hat{c}}_{m}\left(t\right)$ are the MFCCs of the synthesized or processed speech at time frame t,
- T is the total number of time frames,
- M is the number of MFCC coefficients used in the comparison (typically 12 or 13),
- ln(10) is the natural logarithm of 10, used for scaling the result into decibels (dB).

### 2.3 Predictive model development and validation

To develop a unified objective metric, we trained supervised learning models to predict the subjective MOS scores using the objective STOI and MCD values as input features.

*1) Model Training and Comparison:* We compared the performance of three different regression models:

- **Piecewise Linear Model:** Our original baseline model, which uses a heuristic entropy threshold to split the data.
- **Support Vector Regressor (SVR):** A standard non-linear model with a radial basis function (RBF) kernel.
- **Random Forest Regressor:** An ensemble model consisting of multiple decision trees.

*2) Validation:* To ensure generalizability and prevent overfitting, we employed a rigorous Leave-One-Dataset-Out (LODO), or Leave-One-Trial-Out (LOTO) for the piecewise linear model, cross-validation scheme. In each fold of the validation, one of the 10 datasets (or trials) was held out as the test set, and the models were trained on the remaining nine datasets. This process was repeated 10 times, with each dataset serving as the test set once. The final performance metrics were calculated on the aggregated out-of-sample predictions from all 10 folds.

### 2.4 Statistical testing

All statistical testing in this work is conducted using *SciPy* (“SciPy 1.0: Fundamental Algorithms for Scientific Computing in Python”, 2020), with the significance level set to .05. Performance of the regression models was evaluated using the coefficient of determination (R²) and Mean Absolute Error (MAE).

## 3 Results

**Fig. 2.**
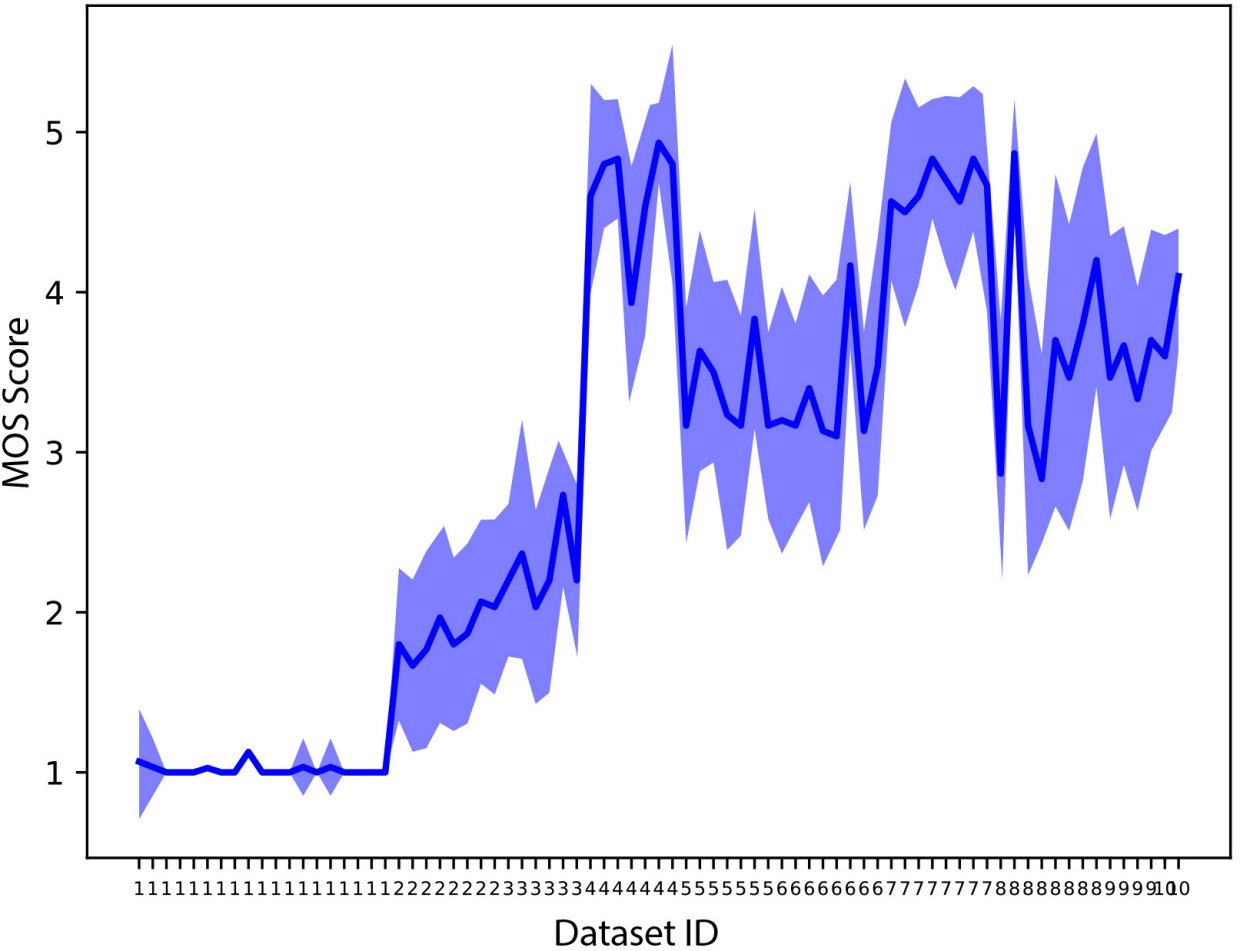
MOS scores. Each numerical value of the x-axis represents an audio sample, which is grouped by dataset (e.g, 19 ‘1’s on the x-axis correspond to 19 samples from dataset 1). The y-axis is the MOS score. The bold blue line represents the mean value while the shaded area indicates the mean ± standard deviation.

The MOS scores obtained from 14 evaluators are presented in Figure 2. These scores serve as the perceptual ground truth for evaluating the objective metrics. There is a clear variation in perceived quality both within and across the 10 datasets.

### 3.1 Issues with individual method

**Fig. 3.**
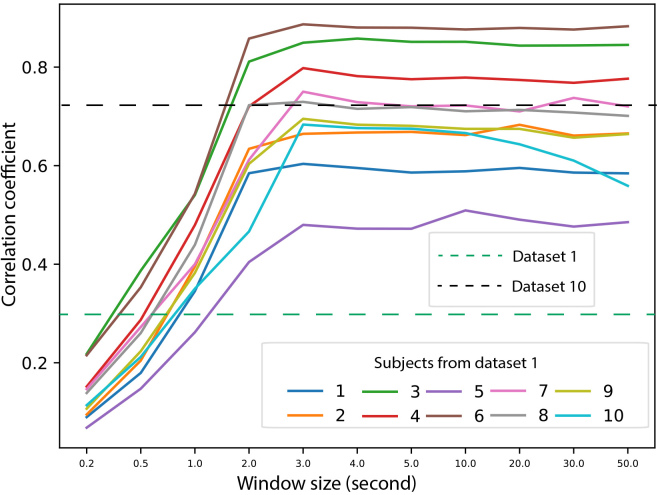
This figure demonstrates how the correlation coefficient changes with various window sizes. Different colors represent different subjects in dataset 1. Mean CC for datasets 1 and 10 are also plotted as two horizontal dashed lines. Note that CC of dataset 1 is incorrectly higher than dataset 10 using long window.

*1) Correlation Coefficient (CC):* This section investigates how CC is influenced by the length of audio files using dataset 1. Audio with different lengths, ranging from 0.2 s to 50 s, are taken from the 10 audio files, and CCs are calculated, as presented in Figure 3. In addition, the average CC of datasets 1 and 10, with a length of 0.3 s, are plotted as the dotted lines.

This analysis demonstrates that CC scores are highly sensitive to the analysis window size, making comparisons between studies that use different parameters unreliable.

**Fig. 4.**
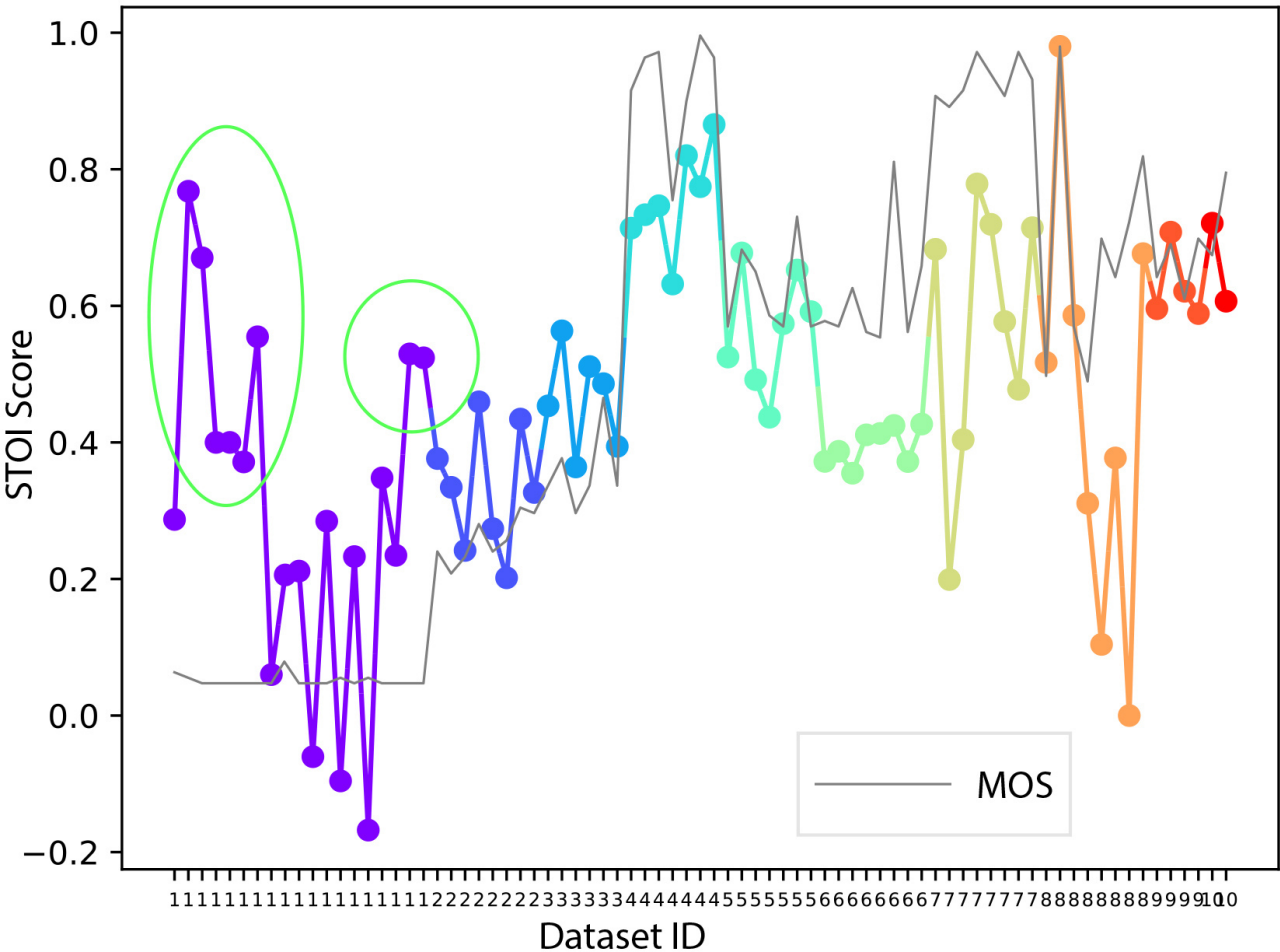
STOI and MOS of individual trials from all 10 datasets. However, the STOI scores of several trials from dataset 1 are overrated, as indicated by the green circles, which contradict the MOS scores.

*2) Short-Time Objective Intelligibility (STOI):* The STOI scores of all trials from all 10 datasets are presented in Figure 4. However, in this plot, STOI scores from dataset 1 do not align well with the scores in Figure 2. Specifically, STOI scores of several trials from dataset 1, indicated as green circles in Figure 4 are unexpectedly high, even higher than two trials from dataset 10.

**Fig. 5.**
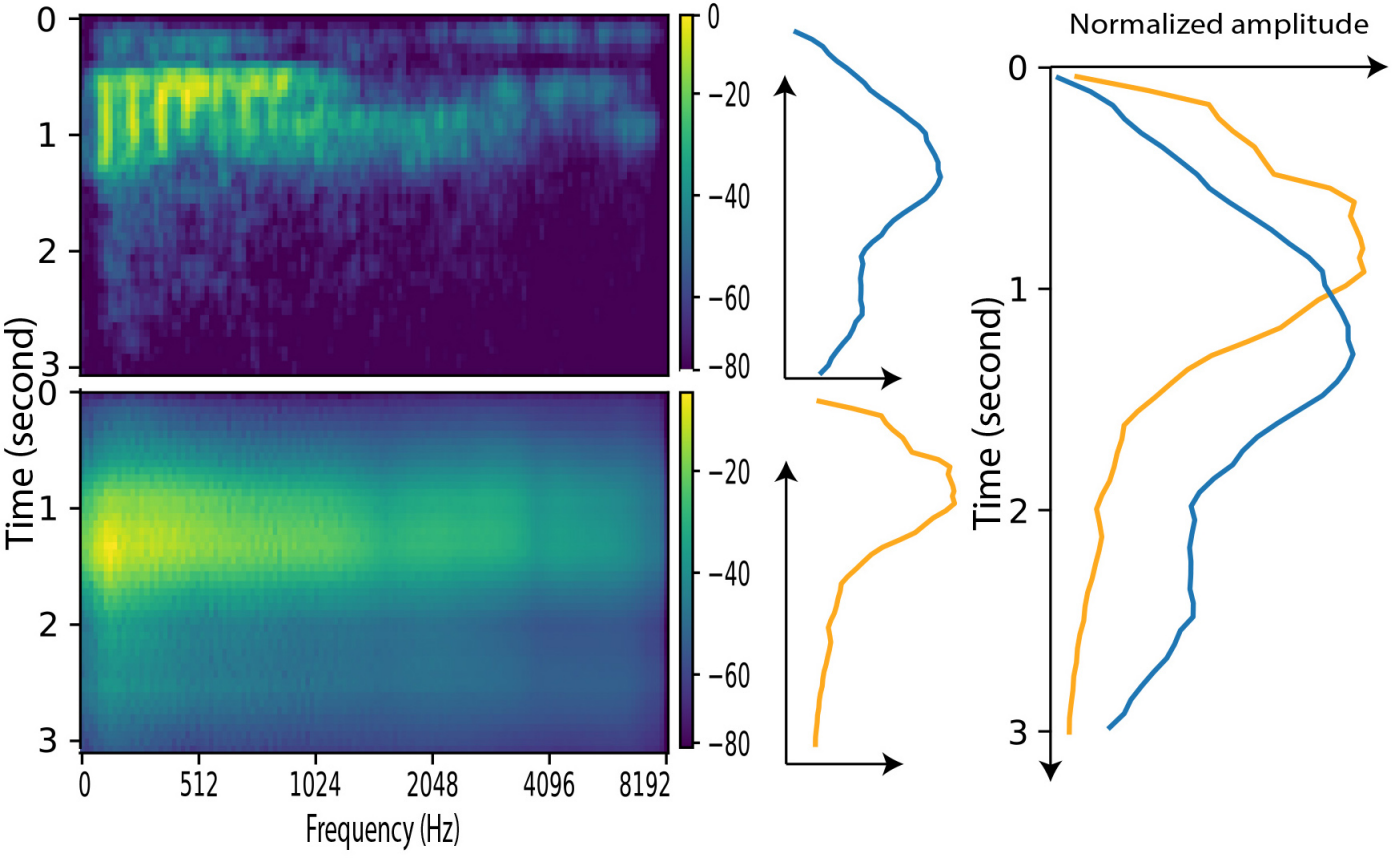
Spectrogram analysis on the trial which has the highest STOI scores from dataset 1. The left subplot represents the spectrogram of the original (top) and the reconstructed audio (bottom). The middle two subplots are generated by averaging the left spectrogram along the frequency axis. To better visualize these two average lines, these two lines are overlapped and displayed in the right subplot.

To investigate this issue, the spectrogram of the original and reconstructed audio corresponding to the highest STOI score in dataset 1 is calculated and displayed in Figure 5. The left subplot represents the spectrogram of the original (top) and the reconstructed audio (bottom). The middle two subplots are generated by averaging the left spectrogram along the frequency axis. To better visualize these two average lines, these two lines are overlapped and displayed in the right subplot. The right subplot shows that the average amplitude oscillations of the two audio signals are very similar. The task is very simple in this situation, which is a single word with a monosyllable production. Therefore, the distribution of amplitude along the frequency axis is simple, and both resemble the bell shape, therefore, the STOI between these two bell-shaped sequences could be high.

**Fig. 6.**
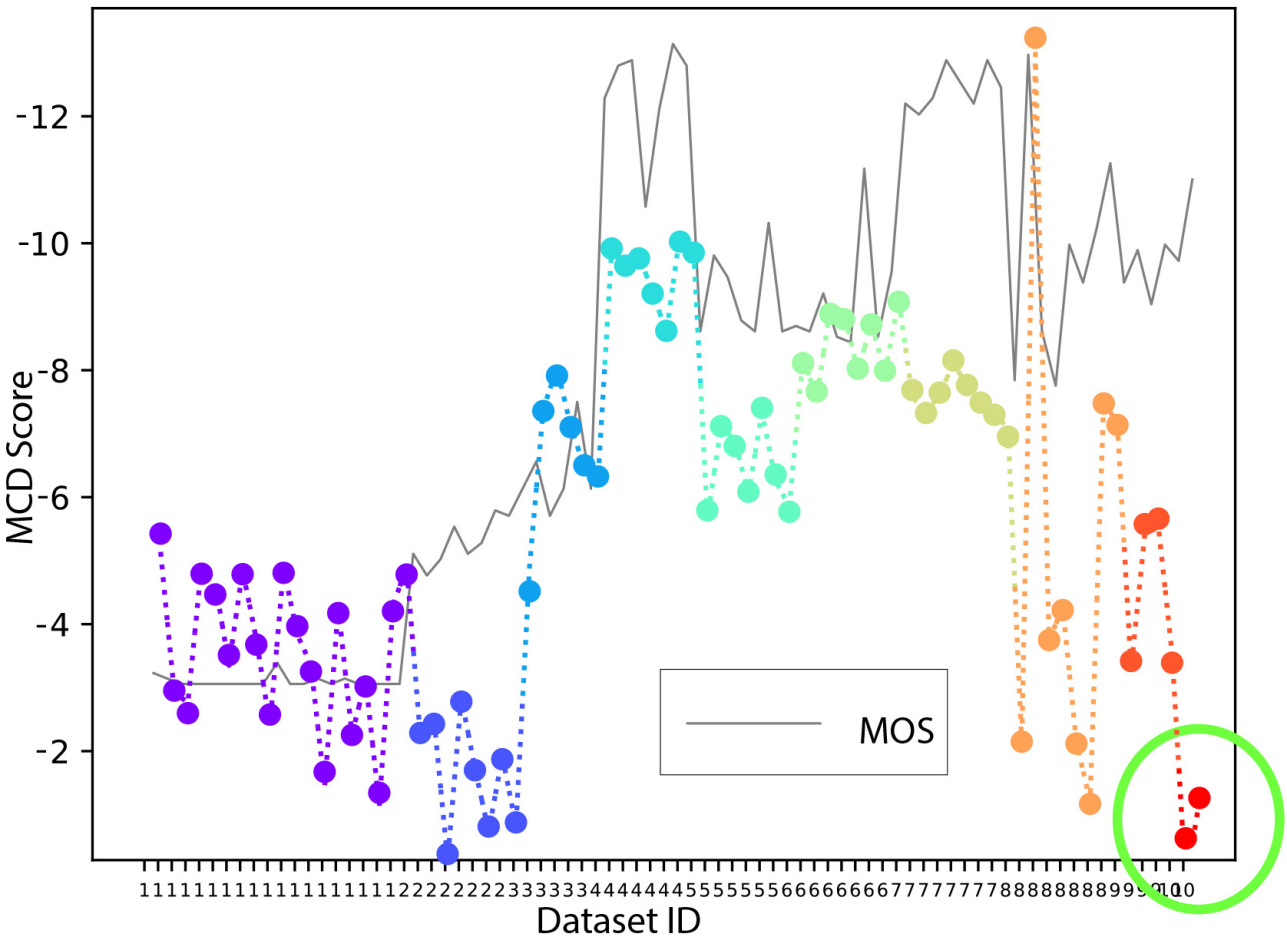
Negative MCD and the shift-aligned MOS score of all trials from 10 datasets. Plotting negative MCD allows for a more intuitive comparison with MOS, where higher values indicate better quality. Note that MCD alone fails on some trials (e.g., from dataset 8, 9, and 10), which have high MOS scores but low negative MCD scores. The green circle highlights two example samples from dataset 10 that exhibit a clear difference in MOS and MCD scores.

From the previous analysis, it is clear that STOI could be high even for a bad waveform reconstruction with a very simple spectral amplitude distribution, such as those from dataset 1. In this situation, MCD should be considered. For example, in Figure 6, all trials from dataset 1 show high MCD, which reflects the lower MOS scores.

*3) Mel Cepstral Distortion (MCD):* This section calculates MCD scores for all trials from 10 datasets and displays them in Figure 6. For a better visualization, the negative MCD scores are plotted and MOS score are shifted to align with the MCD score. MCD is a type of distance-based measurement, therefore, high MCD indicates low quality. However, the MCD scores of dataset 10 are unexpectedly high, especially for the example two trials marked by the green circle in Figure 6.

**Fig. 7.**
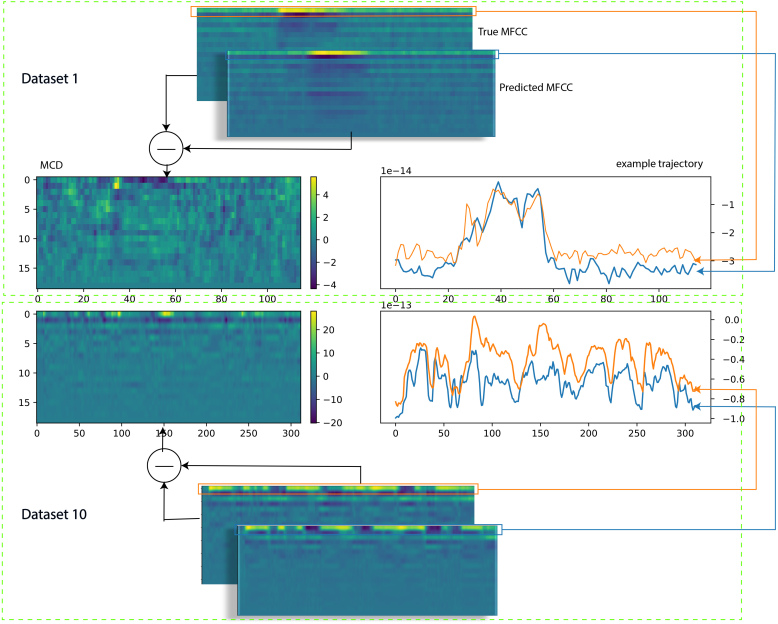
This figure tries to explain the discrepancy between MCD and STOI. The top and lower halves represent datasets 1 and 10, respectively. MCD (middle left) is calculated as the difference between true and predicted MFCC (upper subplot). The two lines (green and blue) in the middle right 1D plots represent the highest frequency component of MFCC from the original and the reconstructed waveform, respectively.

From Figure 4 and Figure 6, it seems that STOI and MCD are not optimal by themselves, and both have shortcomings: STOI fails on dataset 1 and MCD fails on dataset 10. This can be explained by investigating the calculation procedure used by these two measurements, as depicted in Figure 7.

In Figure 7, the calculation procedures of STOI and MCD are investigated. Taking dataset 1 for instance (upper half), the MCD (middle left) is calculated as the difference between the true and predicted MFCC (upper subplot). For the calculation of STOI (middle right), amplitude trajectories of each frequency (represented by the orange and blue lines) are taken as input to produce a correlation score before averaging. The calculation for dataset 10 (lower half) follows the same procedure. Note that MCD is higher for dataset 10, indicated by the color bar.

From this plot, both trials from the two datasets have similar high STOI, as indicated by the high co-oscillation between the original and the reconstructed frequency features (right half subplot). However, the MCD could be very different, as indicated by the legends in the right two subplots: The MCD of dataset 1 ranges from -4 to 4 while the MCD of dataset 10 ranges from -20 to 20. It can also be indicated by a larger difference between true and predicted MFCC trajectories from dataset 10 as depicted in the right half subplot. The contrary situation of high STOI (high quality) and high MCD (low quality) in dataset 10 can arise when the trajectories of the frequency feature of two audio follow similar patterns but differ a lot in absolute measurement. On the other hand, the situation of high STOI (high quality) and low MCD (high quality), as in dataset 1, can arise when not only do the trajectories of the frequency feature of two audio follow similar patterns but also are very close in absolute measurement.

In summary, MCD, a distance-based metric, can unfairly penalize reconstructions that are perceptually good but have a constant spectral offset, resulting in low ratings (high MCD) for high-quality audio (e.g., some trials from Dataset 10 as shown in Fig. 6).

### 3.2 A unified metric via predictive modeling

In our previous discussion, it was demonstrated that both correlation-based and distance-based methods have some limitations, and neither of them can be used alone to capture the true reconstruction performance. On the other hand, both of them can only be used in one circumstance, but not the other. Therefore, in this study, we propose a heuristic piecewise linear model as a simple, interpretable baseline, and several non-linear alternatives, including a Support Vector Regressor (SVR) and a Random Forest Regressor to predict MOS scores from STOI and MCD. The performance of the different models, evaluated using the LODO cross-validation scheme, is presented in Table 2.

**Table 2.** Model performance comparison (LODO cross-validation).

Model	R² score	MAE
Piecewise linear (baseline)	0.675	0.547
Support vector regressor (SVR)	0.832	0.323
Random forest regressor	0.892	0.223

**Fig. 8.**
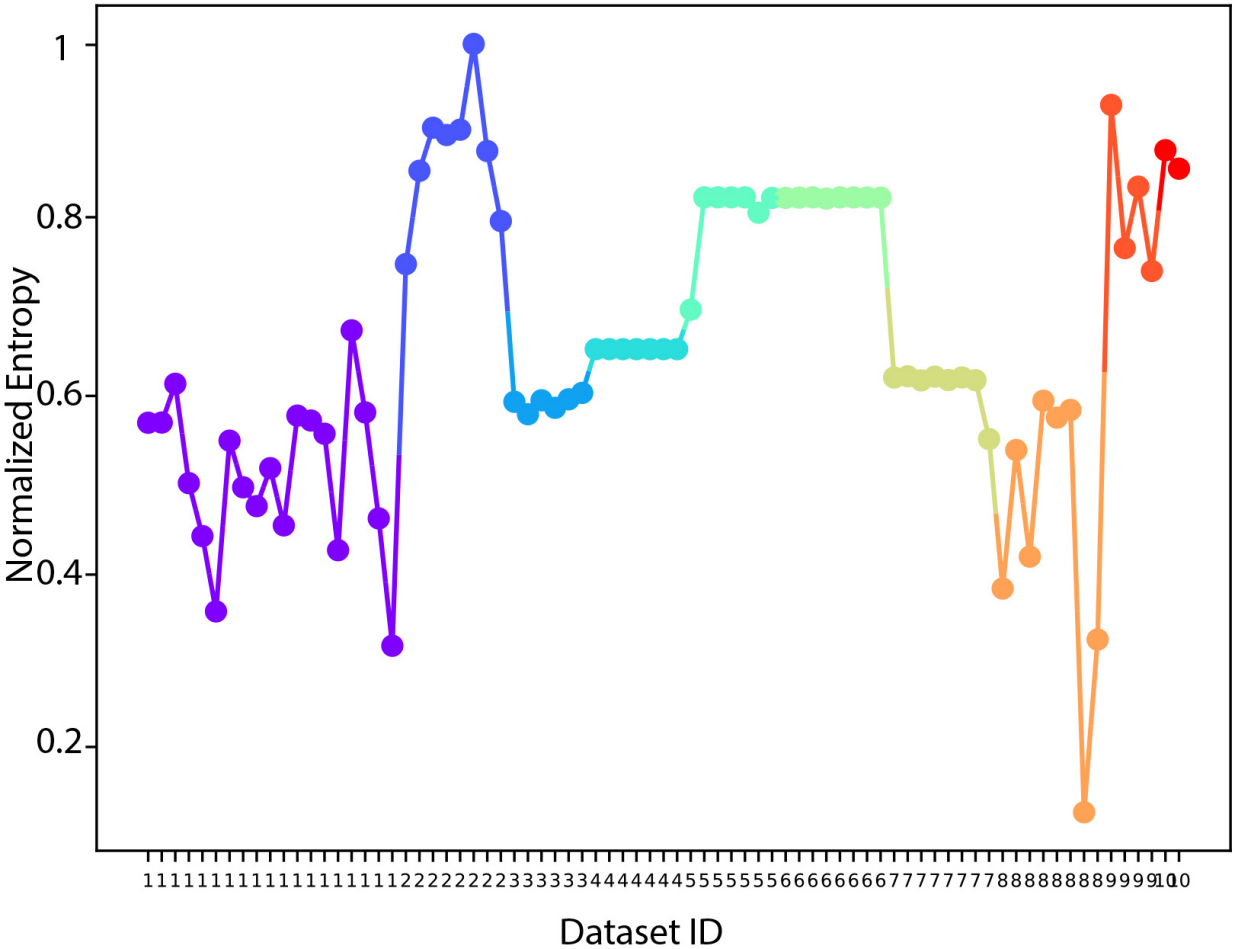
Shannon entropy of all trials from all 10 datasets. The Shannon entropy is used to evaluate the complexity of the signals: simple signals have lower entropy, while complex signals have higher entropy.

The piecewise linear model is inspired by the fact that, as demonstrated in previous sections, STOI and MCD can be misleading when the waveform is simple and complex, respectively. Therefore, we heuristically use Shannon entropy to reflect the complexity of the waveform. The complexity of each audio file is presented in Figure 8.

For the piecewise model, to find the best linear model under these two situations, and because of the limited number of dataset after the partitioning into to situations which makes the leave-one-dataset-out cross-validation impossible, all wave files from 10 datasets are pooled together and divided into two groups according to their entropy value. Then, one linear model is fitted for each group, using the least squares error method, as in equation 5. The average R^2^ and Mean Absolute Error (MAE) are obtained using a leave-one-trial-out (LOTO) cross-validation method.

$$\begin{array}{c} \begin{array}{c} Score=0.101+0.544\times MCD+0.089\times STOI\\ \left(entropy<0.7\right)\\ Score=0.921-0.892\times MCD+0.223\times STOI\\ \left(entropy>0.7\right) \end{array} \end{array}$$ (5)

The entropy threshold is set to 0.7 using a trial-and-error method to achieve the lowest squared error between MOS and model prediction.

**Fig. 9.**
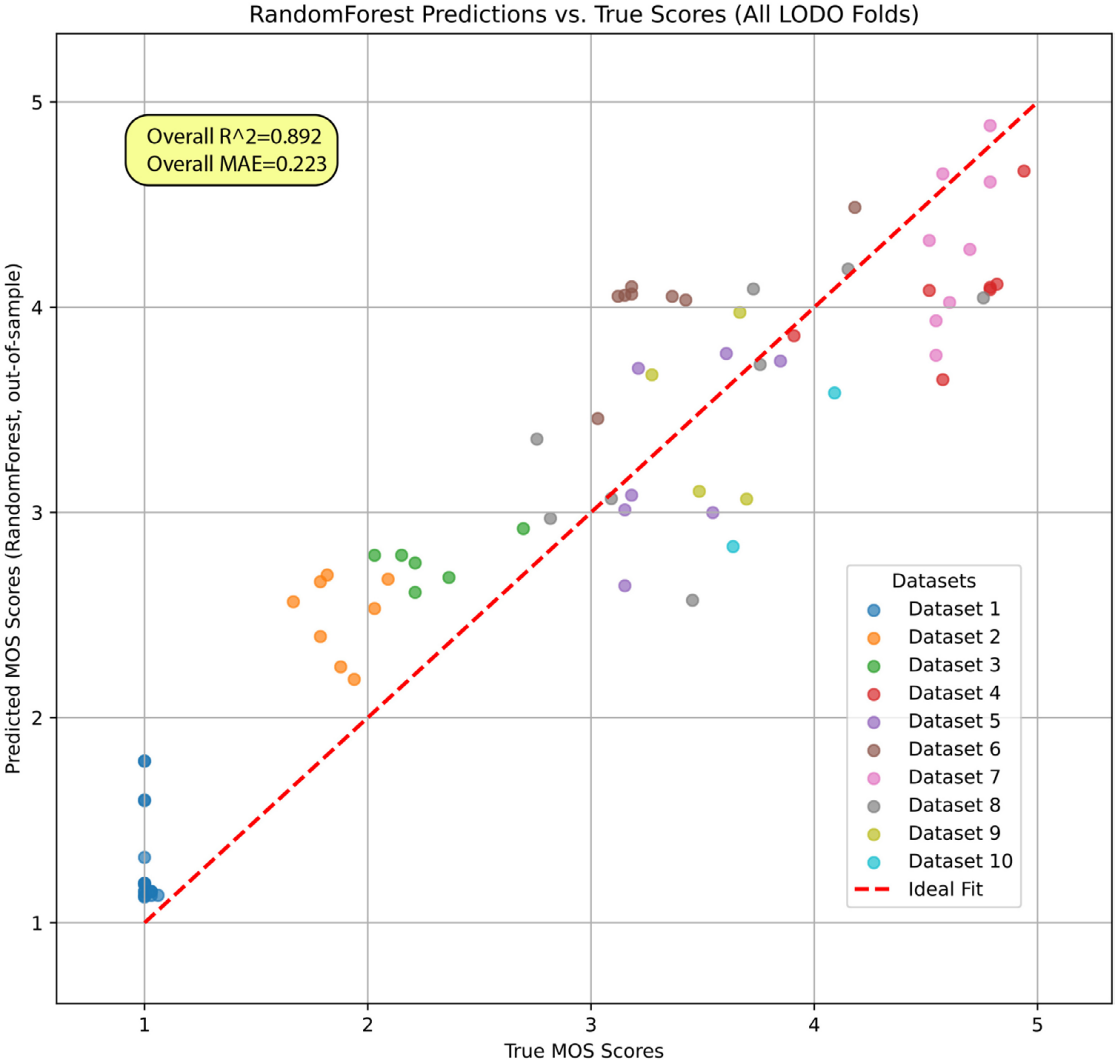
Random Forest model predictions vs. True MOS scores. The plot shows the out-of-sample predictions from the Leave-One-Dataset-Out cross-validation. The model achieves an excellent fit (R² = 0.892) to the true MOS scores across all 10 datasets, demonstrating its robustness and generalizability.

The results in Table 2 clearly indicate that the Random Forest Regressor provides the most accurate prediction of the ground-truth MOS scores. Its performance significantly surpasses the other models. The strong fit of the Random Forest model is visualized in Figure 9, which plots the out-of-sample predicted MOS scores against the true MOS scores for all trials.

## 4 Discussion

### 4.1 Summary

A comprehensive literature review is conducted, and it is found that although many studies have been conducted to reconstruct speech directly from the intracranial signals, there is a lack of a standard evaluation method, making cross-study comparisons difficult. To close this gap, several different objective evaluation methods of waveform reconstruction in speech BCIs are evaluated, including correlation coefficient (CC), short-time objective intelligibility (STOI), and mel cepstral distortion (MCD). 10 publicly available datasets are used during the evaluation, and several shortcomings are found for each of these three methods.

Before evaluating each objective measurement, a subjective measure, mean opinion score (MOS), is produced from 14 human volunteers, which acts as the perceptual reference for the objective evaluation. Then, each objective measurement is evaluated using the above 10 datasets.

Finally, our study demonstrates that while individual objective metrics like STOI and MCD have significant limitations, they can be combined within a data-driven model to create a robust and reliable evaluation metric for speech BCI research. The proposed Random Forest model, which maps STOI and MCD to a predicted MOS score, provides an excellent approximation of human perceptual judgments. One significant advantage of this new metric is that it enables meaningful cross-study comparison. By providing a single, validated score that correlates highly with subjective quality, researchers can now benchmark their systems against a common standard.

In summary, a lack of standard is identified in the literature about waveform reconstruction in speech BCIs, and this study proposes the combination of STOI and MCD as the optimal measurement to accurately reflect the decoding quality and enable cross-study comparison.

### 4.2 Direct speech decoding in BCIs

Direct Speech Decoding in BCIs, often referred to as speech BCIs, aims to directly reconstruct waveforms from neural signals. Almost all studies would first decode speech features, such as spectrograms (Angrick et al., 2019; Metzger et al., 2023; Willett et al., 2021), before converting them into speech waveforms using algorithms such as Griffin-Lim (Verwoert et al., 2022; Wu et al., 2024) or deep learning-based methods (WaveNet (Angrick et al., 2019), LPCNet (Angrick et al., 2024), WaveGan (Berezutskaya et al., 2022), Waveglow (Kohler et al., 2021) etc.).

During the decoding of speech features, a small window is used to slide along neural signals, and waveform features are decoded at each step, then the final features are produced by concatenating previous results. Therefore, the evaluation methods used in speech BCIs are very different from other domains, such as audio transmission or signal processing. In speech BCIs, besides some measurements that are commonly used in other domains, such as STOI, MCD, etc., there are other aspects that are equally important to evaluate the decoding performance:

- Voice activity detection (VAD). In speech BCIs, it is a common practice to employ a two-step approach: first, a binary classifier is used to detect speech activity, then a second regression algorithm is triggered to reconstruct speech step by step if the previous step detects speaking activity, otherwise no speech will be decoded (Angrick, Ottenhoff, Diener, et al., 2021; Angrick et al., 2024; Berezutskaya et al., 2022; Bocquelet et al., 2016; Luo et al., 2023; Moses et al., 2018, 2021). Therefore, besides evaluation of the reconstructed waveform, it is equally important to assess how accurate the speech activity is detected.
- Prelinguistic information. One important target of speech BCIs and their advantage over text BCI, which decode discrete text, is their preservation of prelinguistic characteristics of patients, such as tone, emotion, and gender, which convey a lot of context information about the conversation. Therefore, it is also important for speech BCIs to evaluate the prelinguistic information. For example, in one study, after the waveform is reconstructed, multiple listeners are required to listen to the waveform and identify the gender (Akbari et al., 2019). In other studies, formants and timbre are the decoding targets which are essential to preserve the acoustic characteristics (Anumanchipalli et al., 2019; Bouchard & Chang, 2014; Brumberg et al., 2010).

From the above analysis, it is obvious that the evaluation of speech reconstruction is a comprehensive process that should consider numerous aspects, each of which reflects different aspects of waveform reconstruction.

### 4.3 Limitations

We acknowledge the inherent limitations of using MOS as a ground truth, including potential subjective biases and ceiling effects on simpler tasks. However, the high inter-rater reliability observed in our study (Cronbach’s alpha = 0.8687) provides confidence in the stability of our target variable.

Furthermore, besides what is described in this work, there are many other standard speech quality metrics employed in other areas, such as the Perceptual Evaluation of Speech Quality (PESQ) and Dynamic Time Wrapping (DTW). Considering the uniqueness of audio reconstruction in speech BCI and the validity of PESQ or DTW for these unique BCI-specific distortions cannot be assumed, which would require its own dedicated evaluation study, we will make this investigation of other methods our future work.

## 5 Conclusion

This work identifies critical issues with existing evaluation methods in waveform reconstruction for speech BCIs, which currently prohibit robust cross-study comparisons. We demonstrated that commonly used objective metrics like CC, STOI, and MCD have individual shortcomings. To address this, we developed a unified metric by training models to predict human-rated Mean Opinion Scores (MOS). Through a rigorous leave-one-dataset-out cross-validation, we found that a Random Forest regressor that uses STOI and MCD as inputs provides the most accurate and generalizable prediction of perceived audio quality. We propose the use of this validated model as a standardized evaluation tool to enable more reliable benchmarking and foster cumulative progress in the development of speech neuroprosthesis.

## Data Availability

The datasets analyzed during the current study are available from the original publications as cited in Table 1.
